# Psychometric properties of KIDSCREEN-27 among childhood cancer survivors and age matched peers: a Rasch analysis

**DOI:** 10.1186/1477-7525-11-96

**Published:** 2013-06-13

**Authors:** Anna Jervaeus, Anders Kottorp, Lena Wettergren

**Affiliations:** 1 Department of Neurobiology, Care Sciences and Society, Division of Nursing, Karolinska Institutet, Stockholm, Sweden; 2 Department of Neurobiology, Care Sciences and Society, Division of Occupational Therapy, Karolinska Institutet, Stockholm, Sweden

**Keywords:** Health-related quality of life (HRQoL), Kidscreen, Psychometrics, Rasch analysis

## Abstract

**Background:**

There is a growing population of children and adolescents that have survived their cancer diagnosis. Therefore, it is of great importance to perform follow-up studies with relevant, valid and sensitive measures. It is of interest both to follow changes over time and to compare results from childhood cancer survivors with those from persons without this experience, to fully understand the impact and complexity of childhood cancer in regard to different aspects of quality of life. The aim of this study was to evaluate the psychometric properties of KIDCSREEN-27 for use with survivors of childhood cancer.

**Methods:**

KIDSCREEN-27 consists of five dimensions measuring health-related quality of life (HRQoL) in children and adolescents; 63 survivors, (4–6 years post- diagnosis) aged 12–22 and 257 from a comparison group were assessed. KIDSCREEN-27 was evaluated using a Rasch Partial Credit Model (PCM). The aspects studied were the properties of the rating scale including threshold values, internal scale validity, unidimensionality, person response validity, and differential item functioning (DIF) comparing the survivors with peers.

**Results:**

The rating scales revealed almost expected patterns of responses, and the threshold ordering for two of three rating scales displayed acceptable results. The items demonstrated acceptable goodness-of-fit *MnSq* values in 23 of 27 items (85.2%). The explained variance within each dimension was above the set criterion (50%) for all dimensions except Autonomy & Parent Relations (39.8%). Person goodness-of-fit showed acceptable results in four of five dimensions. No DIF was detected with regard to cancer experience (survivors/comparison group).

**Conclusions:**

Based on the performed Rasch analysis, KIDSCREEN-27 is recommended, with the exception of Autonomy & Parent Relations, due to non-satisfactory unidimensionality, for use among adolescents and young adults who have survived childhood cancer. Still, it is recommended that future research should include a larger sample of childhood cancer survivors in order to monitor some items more thoroughly and explore different levels and patterns of HRQoL in KIDSCREEN-27.

## Background

Several large cohort studies have shown that long-term survivors of childhood cancer are at high risk of developing serious health problems [1,2] and this risk increases with time [1]. Interestingly, self-reported HRQoL or quality of life (QoL) among long-term survivors has been shown to be almost equal to or higher, than that of controls [3-5]. Survival rates have improved remarkably over recent decades, and survival probability at ten years among those diagnosed with cancer in childhood, is approximately 75% [6]. This means that society has a growing population of long-term childhood cancer survivors, and a significant proportion of them have chronic health conditions. It is of great importance to follow HRQoL among survivors, particularly since there seems to be a discrepancy between clinical health outcomes and the self-reported HRQoL.

In a European collaboration project, researchers have developed the KIDSCREEN instruments, which are designed for the assessment of HRQoL in both chronically ill and healthy children and adolescents, aged 8–18 years [7]. HRQoL is described as a multidimensional concept, elucidating respondents’ own views regarding their health state, and should include aspects of physical, mental and social health [8]. The developmental process, which included literature reviews, expert consultation, and focus groups with children and adolescents as well as their families in the 13 participating European countries, resulted in three versions of the instrument [7]. The three versions differ in length and included dimensions. KIDSCREEN-52 provides detailed information within ten HRQoL dimensions, KIDSCREEN-27 is a shorter version of KIDSCREEN-52 in which the ten dimensions are summarised into five dimensions. Finally, KIDSCREEN-10 was developed from the 27-version and provides one global HRQoL-score [7]. Determination of the degree of accordance between corresponding dimensions in the 27-version and the longer 52-version have shown coefficients ranging from r = 0.63 to r = 0.96 [9].

Internal consistency, as measured by Cronbach’s Alpha, has shown acceptable results for the KIDSCREEN-27 [10,11] and KIDSCREEN-52 [12,13], as has test-retest reliability, for both versions [9,13]. Regarding construct validity, investigations of convergent validity, measured by correlations between the KIDSCREEN dimensions and other HRQoL measures assessing similar aspects, have shown moderate to high correlation coefficients for both the −27 and the −52 versions [9,12,13]. Furthermore, confirmatory factor analysis has shown that most dimensions fit data well for both the −27 [11] and the −52 versions [12-14]. Analyses of outcomes in relation to socioeconomic status and health problems have shown socioeconomic status to have a positive association with most of the dimensions for the −27 version [9] and for all in the −52 version [13]. Additionally, statistically significant differences have been found within all dimensions, in both versions, between children with and without physical and mental health problems, whereby those with health problems showed lower mean values compared to those without health problems [9,13].

Aspects of the Rasch model have been used in a few studies [11,13-15]. The results have generally been promising regarding the KIDSCREEN instruments, both from a developmental point of view as well as regarding usage among children and adolescents, both healthy and with cerebral palsy [14]. However, as evidence of validity of an instrument is sample dependent it is of great importance to perform more in-depth validity studies with different target groups, e.g. childhood cancer survivors in this study, as specific psychometric issues in certain groups may not be detected in large population studies. To our knowledge, some studies have been published regarding the clinical usage of KIDSCREEN in children with cancer or tumour experience [16-19], but so far no results have provided evidence of the validity of the KIDSCREEN measures in relation to children and adolescents with cancer experience.

There is a growing population of children and adolescents that have survived their cancer diagnosis. Therefore, it is of great importance to perform follow-up studies with relevant, valid, and sensitive measures in order to make comparisons among children and adolescents by subgroups (sex, age, diagnoses). It is of interest both to follow changes over time and to compare results from childhood cancer survivors with those from persons who have not experienced cancer, to fully understand the impact and complexity of childhood cancer in regard to different aspects of quality of life. Furthermore, it is of value to find a reliable instrument to be able to use as a screening tool for identifying those survivors in need of extra support. Even though the KIDSCREEN instruments have been psychometrically tested, using classical test theory and to some extent also Rasch, it’s robustness among survivors of childhood cancer has not been investigated, which could be of importance due to a growing number of survivors in society. Do the actual data patterns support the assumption of an underlying construct from an item as well as a person perspective? Taking the above factors into account, the aim of this study was to evaluate the psychometric properties of the five dimensions in KIDCSREEN-27 for use in survivors of childhood cancer. The specific research questions were:

1. What are the psychometric properties of the different rating scales used in KIDSCREEN-27?

2. Is there satisfactory evidence of internal scale validity and person response validity in the generated KIDSCREEN-27 measures?

3. Is there evidence supporting unidimensional underlying constructs within the different dimensions?

4. Do the items in KIDSCREEN-27 function in the same way, indicated by no presence of differential item functioning (DIF), among childhood cancer survivors compared to a comparison group?

## Methods

### Sample

#### Survivors

Our research group followed a national cohort of school-aged children that had been diagnosed with cancer between 2004 and 2006 [20]. This report concerns a follow-up, performed in 2010, of the group at a median of 63 months (range 50–74 months) after diagnosis. Among the eligible survivors (N = 92), 63 agreed to participate (response rate 68%), median age 17, range 12–22 years. The diagnostic groups were: acute lymphoblastic leukaemia (n = 21); skeletal and soft tissue sarcoma (n = 15); tumours of the central nervous system (CNS) (n = 10); Hodgkin’s lymphoma (n = 6); non-Hodgkin’s lymphoma (n = 6); acute myeloid leukaemia (n = 3); other diagnoses (n = 2) (one Sertoli/Leydig cell tumour and one germ cell tumour).

#### Comparison group

Participants (N = 500) were randomly selected from the Swedish population register (SPAR), to resemble the study group regarding age. From the 500 eligible participants, 24 were excluded due to being abroad (n = 10), insufficient knowledge of the Swedish language (n = 5), unidentifiable address (n = 5), cognitive dysfunction (n = 3) and prior cancer experience (n = 1). Finally, 257 (54%) agreed to participate, median age 16, range 11–23, and 219 declined to participate, either actively (n = 171) or passively, e.g. they did not respond to letters (n = 48). The comparison group was, in this study, only used for the DIF analyses.

### Measures

KIDSCREEN-27 consists of five dimensions: Physical Well-being, Psychological Well-being, Autonomy & Parent Relations, Social Support & Peers, and School Environment. The items follow a 5-point Likert-type scale [13] with three different sets of responses: i) poor, fair, good, very good, excellent; ii) not at all, slightly, moderately, very, extremely; iii) never, seldom, quite often, very often, always. Respondents are asked to answer the question in relation to previous week. Four items, negatively formulated, were re-coded according to standard procedures [7].

### Procedure

Approval for this study was obtained from the Regional Ethical Review Board in Stockholm.

An information letter was sent to all eligible participants. For those who agreed to participate a suitable time for a telephone-administered interview (KIDSCREEN-27) was agreed upon, and for those who preferred to answer the questionnaire at home, a questionnaire was sent by mail (3% study group; 11% comparison group). For the survivors, written informed consent was obtained from the participants and from parents when participants were under 18 years of age. For the comparison group, written informed consent was obtained from participants, and from parents for those under 18. For those over 18, verbal consent was obtained directly before the telephone-administered questionnaire was answered. Reminder letters were sent to those who were difficult to reach. All participants received a cinema ticket as a form of incentives.

### Data analyses

Descriptive statistics (demographics) were calculated using the IBM® SPSS® Statistics Version 20.

The Rasch approach offers a method of simultaneously generating measures for persons related to their ability, and items related to their difficulty [21] based on ordinal data. The approach is being increasingly used in health sciences research with the intention of developing and examining the measurements used [22]. The construct of KIDSCREEN’s five dimensions was evaluated using a Partial Credit Model (PCM), a Rasch model designed for polytomous data [21]. The Rasch analysis software program WINSTEPS®, version 3.72.2 and 3.73 [23], was used to perform the Rasch analysis.

For the dimensions of Physical Well-being, Psychological Well-being, Autonomy & Parent Relations and Social Support & Peers, 63 survivors of childhood cancer participated. Within the dimension School Environment 49 answered, due to four not attending school in the previous week for reasons such as hospital visits, sickness or trainee. For 10 participants this dimension was not applicable due to work, military service, sick leave or unemployment. For the item “Have you been able to run well?” within Physical Well-being there were 10 (16%) missing responses. As Rasch models are suitable for handling datasets that do contain missing values [21] we did not have to exclude any participant due to missing data in order to use the Rasch modelling procedures.

The rating scales were initially examined by analysing the category structure, expressed by the observed average and outfit mean square values *(MnSq)*. The guidelines set out by Linacre [24] were followed. These recommend that, e.g., (a) all rating scale categories and thresholds should advance monotonically and (b) the rating scale category outfit *MnSq* value should be below 2.0.

Internal scale validity and person-response validity was investigated by item and person goodness of fit statistics. Calculated statistics were represented by Mean Square *(MnSq)* residuals and standardized *z*-values, which indicate to what extent the actual responses from KIDSCREEN-27 match the expected responses in the Rasch model, for items as well as persons. According to Smith, Rush, Fallowfield, Velikova and Sharpe [25] it is preferable to use the *MnSq* statistics for polytomous data as they are less sensitive to sample size, compared to t-statistics. Threshold values, chosen for item *MnSq* infit statistics were 0.6-1.4 for the rating scale (Likert/survey) [21]. As each dimension only contains a limited number of items, we set the criterion that all items within each dimension would fit the Rasch model. For person infit statistics, threshold values were chosen to be < 1.4 (Infit *MnSq*) and < 2.0 (*z*-value) in order to be evaluated as meeting the criteria of acceptable person goodness of fit. Generally, it is accepted that up to 5% of the respondents can show non-satisfactory goodness of fit without threatening person response validity. Additionally, floor and ceiling effects were calculated.

Unidimensionality was measured by monitoring the variance explained for each dimension from KIDSCREEN-27 by the use of a principal component analysis (PCA) of residuals. The criterion was set that the variance explained by measures should exceed 50% [26]. Unexplained variance in 1st contrast, a potential secondary dimension in the data, is generally accepted to be not more than 5% (monitoring multidimensionality).

Uniform DIF was analysed to explore the stability of item difficulty when comparing childhood cancer survivors to a comparison group. The magnitude of uniform DIF was investigated by Mantel-Haenszel statistics [27] (p < 0.01).

## Results

The background characteristics, from both survivors and comparison group, are shown in Table 1.

**Table 1:** Demographic characteristics of participating survivors and comparison group

	**Survivors**	**Comparison group**	**df**	**pª**	**Total sample**
	**n = 63**	**n = 257**			**n = 320**
	**n, (%)**	**n, (%)**			
Sex, n (%)			1	0.092	
Female	26 (41)	139 (54)			165 (52)
Male	37 (59)	118 (46)			155 (48)
Living situation, n (%)					
With parent/parents	56 (89)	214 (83)	1	0.364^b^	270 (84)
Alone	4 (6)	18 (7)			22 (7)
With partner	1 (2)	14 (6)			15 (5)
Other constellation*	2 (3)	11 (4)			13 (4)
Occupation, n (%)					
Education	52 (82)	206 (80)	1	0.802^c^	258 (81)
Work	6 (10)	41 (16)	1	0.274^d^	47 (15)
Unemployed	3 (5)	10 (4)			13 (4)
Sick leave	2 (3)	0			2 (<1)

ªTested for differences in proportions by Chi-square test.

^b^Tested between those living with one or two parents vs. those reporting other living arrangements.

^c^Tested between those in education vs. those not in education.

^d^Tested between those working vs those not working.

*Other constellation: includes living with friends, other relatives or combined living, e.g. parents/student apartment.

### Rating scales/category function

The average measures for the three types of rating scales used in KIDSCREEN-27 advanced in the expected direction, except for response categories 1 and 2 in the rating scale with categories “poor, fair, good, very good, excellent” used only for one item (In general, how would you say your health is?) within the dimension Physical well-being. The outfit *MnSq* values were all below 2.0. The threshold ordering for two of the rating scales displayed acceptable results, but the rating scale “not at all” to “extremely” displayed disordered thresholds between response category 2 and 3. As the problems detected were only related to one response category and a limited number of responses, we chose not to collapse the response categories.

### Internal scale validity

All items showed *MnSq* values within the range (0.6-1.40) except for four items: “Have you felt fit and well?” (0.53), “Have you been able to run well?” (1.60) within the dimension Physical Well-being, “Have your parent (s) treated you fairly?“ (1.62) within Autonomy & Parent Relations, and “Have you been able to rely on your friends?” (1.51) within Social Support & Peers (Table 2).

**Table 2:** Results from the Rasch analysis of the psychometric properites of KIDSCREEN-27 in childhood cancer survivors (n = 63)

	**Physical well-being (5 items) N = 63**	**Psychological well-being (7 items) N = 63**	**Autonomy & parent relations (7 items) N = 63**	**Social support & peers (4 items) N = 63**	**School environment (4 items) N = 49**^**e**^
Number of responses included in the analyses, n	305	441	441	252	195
Possible responses if no missing values (n)	(315)	(441)	(441)	(252)	(196)
Item misfit, n	2^a,b^	0	1^c^	1^d^	0
Variance explained, %	57.7	60.2	39.8	52.6	64.0
Unexplained in 1st contrast, %	13.4	15.1	16.6	18.4	15.7
Person misfit, n (%)	2 (3)	5 (8)	2 (3)	3 (5)	0
Ceiling, n (%)	1 (2)	2 (3)	6 (10)	6 (10)	5 (8)
Floor, n (%)	0	0	0	1(2)	1(2)

^a^Have you felt fit and well?

^b^Have you been able to run well?

^c^Have your parent(s) treated you fairly?

^d^Have you been able to rely on your friends?

^e^N = 49 due to n = 4, not attending school the previous week and n = 10, not applicable.

### Unidimensionality

The explained variance within each measured dimension was >50% for four of the dimensions, Autonomy & Parent Relations displayed a value of 39.8%; the unexplained variance in 1st contrast was above 5% for all dimensions (Table 2).

### Person response validity

Regarding person goodness of fit, the dimensions demonstrated different results. Psychological Well-being displayed a proportion of persons demonstrating values slightly above 5%, while the other four dimensions showed acceptable values at 5% or below (Table 2).

**Figure 1:**
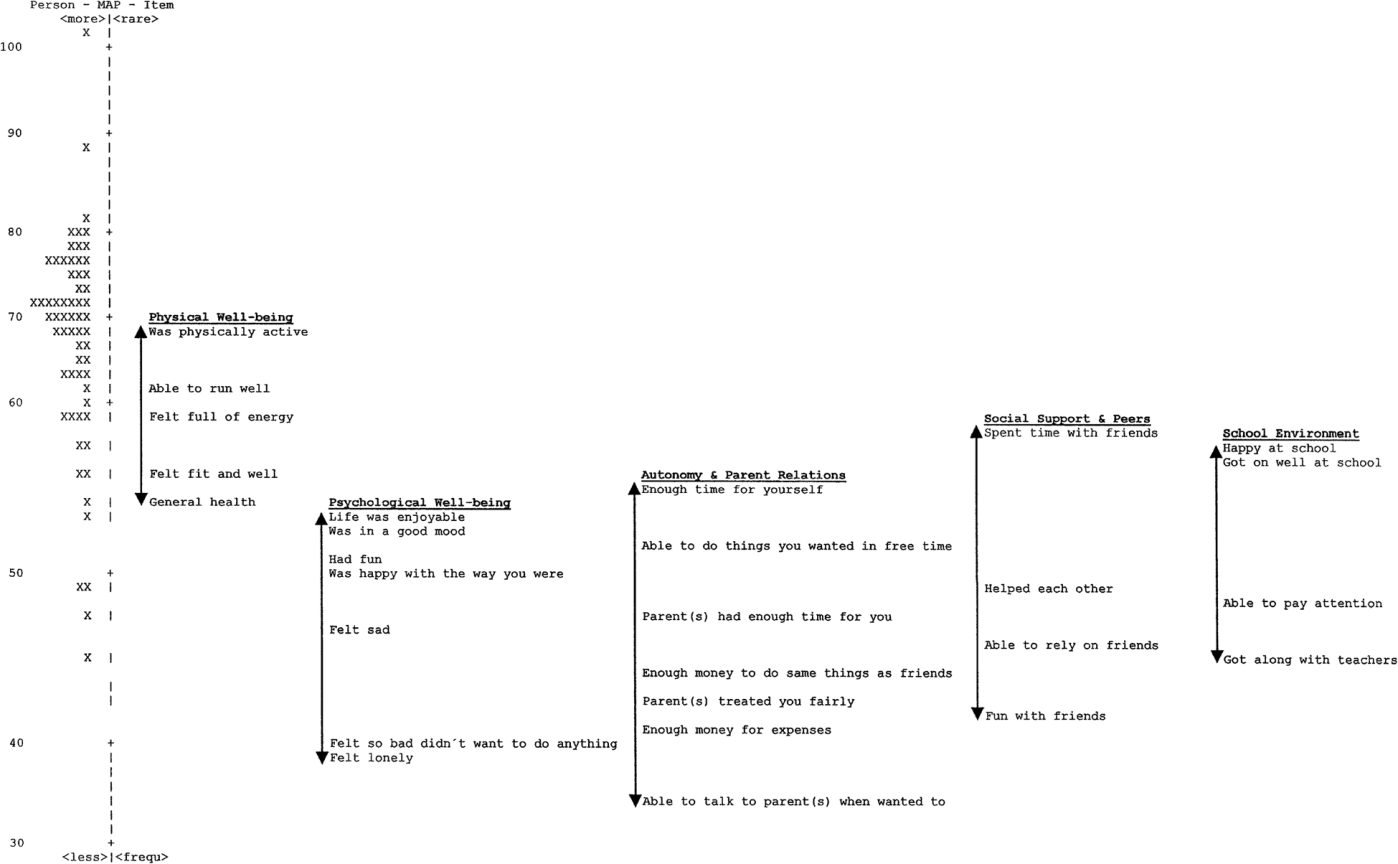
**Person-item map of KIDSCREEN-27.** Persons (left side) versus Items (right side).

Ceiling effects were present for all dimensions and floor effects in two dimensions (Social support & Peers and School Environment) (Table 2). A persons versus items map is shown in Figure 1, displaying the equal interval scale given by the Rasch model [28]. The logit scale is displayed on the far left, and the person measure in the next column, where each ‘X’ represents one person displayed by their ability, or in this case, the level of HRQoL. Item difficulty calibrations or in this case how challenging each item is, are displayed on the right side of the scale. As the participants overall rated their HRQoL high in the included items, the participants are being clustered higher on the scale and the items lower (Figure 1).

### Differential item functioning

No uniform DIF was detected when comparing the childhood cancer survivors with the comparison group.

## Discussion

The aim of this study was to evaluate the psychometric properties of KIDSCREEN-27 with a Rasch analysis in a national cohort of childhood cancer survivors. Overall, the results were satisfactory, with acceptable item goodness-of-fit in 23 of 27 items, acceptable unidimensionality for four of the five dimensions, and acceptable person goodness-of-fit in four of the five dimensions. No uniform DIF was detected between the childhood cancer survivors and the comparison group. With regard to the growing number of survivors in society it is of importance to find a instrument to be able to use as a screening tool at follow up visits and this Rasch analysis could be the first step towards choosing an appropriate instrument. However, given the relatively small sample size (N = 63) the results presented in this paper must be applied with some caution even if it has been suggested that the Rasch model can be used to perform exploratory work with small samples. Based on the results from the Rasch analysis of KIDSCREEN-27 we recommend the instrument to be used among populations of childhood cancer survivors with similar age ranges. Thirty items administered to 30 individuals should have the ability to deliver statistically stable measures, given reasonable targeting and fit [29].

The response categories and threshold disordering that were found were based on a small number of responses/scores, and therefore the number of observations in each rating scale category did not always meet the criterion suggested by Linacre [24]. Taking action (e.g., by collapsing response categories) based upon very few unexpected responses in a small sample may also be inappropriate. If the response categories had been collapsed within this study, it would probably have contributed to an even lower number of misfits, and thus to an improvement of internal scale validity and person response validity. As a small sample may limit the inferences of the fit statistics the findings presented here may actually be underestimating the psychometric performance of KIDSCREEN-27 in a sample of childhood cancer survivors.

Item goodness of fit revealed that 23 of 27 items fitted the model. Three of the items: “Have you been able to run well?” (1.60); “Have your parent(s) treated you fairly?” (1.62); “Have you been able to rely on your friends?” (1.51) displayed underfit to the model, i.e. too much variation in the data, compared to expectations from the Rasch model [21]. These items were all outside the critical range for rating scales (0.6-1.4) but when comparing them to the range for clinical observations (0.5-1.7) [21] all items fitted within the range. It should also be noted that a high proportion (16%) of respondents did not answer one of the items (“Have you been able to run well”). Most of these participants had of different reasons not run the previous week. As the content of these items is relevant for cancer survivors [30,31] we chose not to omit them from the scale, an approach that previously has been used in scale evaluation [32]. It has been stated in the literature that the guidelines regarding fit statistics are supposed to help in detecting problems with items; not just with the decision on which items should be excluded from a test [21]. However, as our criterion was set that no item would display unacceptable goodness-of-fit, the findings in relation to scale validity were mixed. Considering that the sample is fairly small, and previous studies have shown reasonable item fit for both KIDSCREEN-27 [11] and KIDSCREEN-52 [13,14], we need to verify whether these findings are stable with larger samples of cancer survivors, or if they are due to individual variations in this limited dataset. As none of the items did display DIF, when compared to the comparison group, the interpretation of fit statistics is not seen as a major threat to validity, but more a concern to monitor in further studies since the findings do indicate that some individuals score these items differently than expected based on the overall pattern found in the sample. The item “Have you felt fit and well?” showed overfit (less variation) which can indicate redundancy or similar ratings across all participants. As low *MnSq* values may not be a major threat to validity, this item may be of less concern when KIDSCREEN-27 is validated within this sample.

Regarding unidimensionality, the results revealed that the underlying constructs were measured to an acceptable extent, except for the Autonomy & Parent Relation dimension, which showed indications of multidimensionality. Therefore, this dimension is recommended to be further tested among childhood cancer survivors. The possible weakness may have been because this dimension represents a merge of three separate dimensions in the 52-version: Autonomy, Parent Relations & Home Life, and Financial Resources [11]. In contrast, Robitail et al. [11] showed that all five dimensions in the 27-version, for the whole sample (n = 22827), were unidimensional, with regard to infit statistics. They also performed a confirmatory factor analysis that showed acceptable fit to the model. However, an exploratory factor analysis showed that a few items loaded similarly to more than one dimension. Additional analysis, such as PCA of residuals, to measure unidimensionality, was not performed in that study [11]. In the present study the variance explained by the secondary dimension (1st contrast) also showed higher values than the recommended 5% in all dimensions, which can be explained by the fact that there are relatively few items within each dimension in KIDSCREEN-27. The concept of HRQoL has many different aspects [33] and they should measure distinct parts of the concept but still be considered to be interrelated with each other. Qualitative interviews were conducted with the same sample [34], previous to the collection of the questionnaire based data, which revealed results supporting content validity of the KIDSCREEN-27 among childhood cancer survivors.

Person goodness of fit revealed that one dimension (Psychological Well-being) displayed a value above 5% (Table 2). As the number of participants that did not demonstrate acceptable goodness of fit was small, there was no possibility of carrying out more in-depth analyses on subgroup level in this study. On an individual level, no clear pattern was found among the participants that did not demonstrate acceptable goodness of fit; three females and two males, age ranged from 13 to 22 with different diagnoses represented. Future studies with larger sample size would allow for more in-depth explorations, and also for monitoring associations between item and participant misfit. A limited number of responses due to a small sample will also impact on the precision of the item calibration measures. Larger samples will therefore allow for more precise analyses providing evidence of scale validity (e.g., collapsing response categories and exploring residual correlations).

According to the person item map the most challenging dimension was Physical Well-being. The most challenging item was “Was physically active?” and the least challenging item was “Able to talk to parent(s) when wanted to?” It is not surprising that Physical Well-being was the most challenging dimension, since this aspect of HRQoL is the one where impairments and difficulties are expected for the survivors, related to complications because of diagnosis and treatment.

According to the results of the DIF analyses, the items do not appear to work differently for survivors of childhood cancer compared to young people of the same age without a cancer experience. To our knowledge, one previous study has provided results of DIF for KIDSCREEN-27, across different European countries [11], but no study has provided DIF between the sexes, age groups or health status. Regarding KIDSCREEN-52, previous results have shown that none of the items displayed any sizeable DIF by age groups (8–11 vs. 12–18 years), sex or health status [13]. However, in a study comparing children with or without cerebral palsy (CP) some items showed statistically significant DIF; however, this was more frequently seen in the proxy version of the instrument [14]. Based on our findings further validation studies are suggested to explore unique diagnostic profiles in HRQoL, even though this study did not indicate such profiles in relation to survivors after childhood cancer.

An important strength of the present study is that a unique and representative (for five years of survival) national cohort of childhood cancer survivors in Sweden is being followed from 2004 and onwards, with several data collection occasions. However, there are some limitations to the present study that should be mentioned. Firstly, the small sample of survivors of childhood cancer limits the possibility of drawing firm conclusions regarding the robustness of the instrument. Because of the relatively small groups, more sophisticated analyses regarding DIF [22], e.g. for different specific diagnoses, could not be performed. Secondly, as time since diagnosis was relatively short, conclusions regarding the instrument’s performance cannot be drawn for the entire follow-up period after diagnosis. Continued evaluation of the instrument’s psychometric performance in a long-term perspective is recommended, especially as health problems are known to increase over time [1]. Larger cohort studies in a European context would be of value in order to achieve a higher power and also to monitor item and person response validity in more detail. Some participants exceeded the recommended age limits for the instrument of 18 years but no uncertainties were expressed among those older than 18 years when responding to the items.

## Conclusions

Based on the performed Rasch analysis of KIDSCREEN-27, the instrument is recommended, with the exception of Autonomy & parent Relations, due to non-satisfactory unidimensionality, for use among adolescents and young adults who have survived childhood cancer. Still, in relation to the indications of item misfit and multidimensionality for one dimension in this cross-sectional design, it is recommended that future research should include a larger sample of childhood cancer survivors in order to monitor some items more thoroughly and explore different levels and patterns of HRQoL, in KIDSCREEN-27.

## Competing interests

The authors declare that they have no competing interests.

## Authors’ contributions

AJ collected and analysed the data and drafted the manuscript. AK participated in interpreting the data, and helped to draft and revised the manuscript. LW conceived and designed the study, interpreted the data, helped to draft and revised the manuscript. All authors read and approved the final manuscript.

## References

[B1] Oeffinger KC Mertens AC Sklar CA Kawashima T Hudson MM Meadows AT Friedman DL Marina N Hobbie W Kadan-Lottick NS Chronic health conditions in adult survivors of childhood cancer N Engl J Med 2006 355 1572 1582 10.1056/NEJMsa060185 17035650

[B2] Geenen MM Cardous-Ubbink MC Kremer LC van den Bos C van der Pal HJ Heinen RC Jaspers MW Koning CC Oldenburger F Langeveld NE Medical assessment of adverse health outcomes in long-term survivors of childhood cancer JAMA 2007 297 2705 2715 10.1001/jama.297.24.2705 17595271

[B3] Mört S Salanterä S Matomäki J Salmi TT Lähteenmäki PM Self-reported health-related quality of life of children and adolescent survivors of extracranial childhood malignancies: a Finnish nationwide survey Qual Life Res 2011 20 787 797 10.1007/s11136-010-9798-y 21103942

[B4] Mört S Salanterä S Matomäki J Salmi TT Lähteenmäki PM Cancer related factors do not explain the quality of life scores for childhood cancer survivors analysed with two different generic HRQL instruments Cancer Epidemiol 2011 35 202 210 10.1016/j.canep.2010.07.005 20685193

[B5] Sundberg KK Doukkali E Lampic C Eriksson LE Arvidson J Wettergren L Long-term survivors of childhood cancer report quality of life and health status in parity with a comparison group Pediatr Blood Cancer 2010 55 337 343 10.1002/pbc.22492 20582940

[B6] Gustafsson G Heyman M Vernby Å Childhood Cancer Incidence and Survival in Sweden 1984–2005 Book Childhood Cancer Incidence and Survival in Sweden 1984–2005 2007 Karolinska Institutet: City

[B7] The KIDSCREEN Group Europe The KIDSCREEN Questionnaires Quality of life questionnaires for children and adolescents Handbook 2006 Lengerich, Germany: Pabst Science Publishers

[B8] Herdman M Rajmil L Ravens-Sieberer U Bullinger M Power M Alonso J Expert consensus in the development of a European health-related quality of life measure for children and adolescents: a Delphi study Acta Paediatr 2002 91 1385 1390 12578299 10.1111/j.1651-2227.2002.tb02838.x

[B9] Ravens-Sieberer U Auquier P Erhart M Gosch A Rajmil L Bruil J Power M Duer W Cloetta B Czemy L The KIDSCREEN-27 quality of life measure for children and adolescents: psychometric results from a cross-cultural survey in 13 European countries Qual Life Res 2007 16 1347 1356 10.1007/s11136-007-9240-2 17668292

[B10] Erhart M Ravens-Sieberer U Health-related quality of life instruments and individual diagnosis - a new area of application Psychosoc Med 2006 3 1 11 PMC2736504 19742074

[B11] Robitail S Ravens-Sieberer U Simeoni MC Rajmil L Bruil J Power M Auquier P Testing the structural and cross-cultural validity of the KIDSCREEN-27 quality of life questionnaire Qual Life Res 2007 16 1335 1345 10.1007/s11136-007-9241-1 17668291

[B12] Haraldstad K Christophersen KA Eide H Nativg GK Helseth S Health related quality of life in children and adolescents: reliability and validity of the Norwegian version of KIDSCREEN-52 questionnaire, a cross sectional study Int J Nurs Stud 2011 48 573 581 10.1016/j.ijnurstu.2010.10.001 21067750

[B13] Ravens-Sieberer U Gosch A Rajmil L Erhart M Bruil J Power M Duer W Auquier P Cloetta B Czemy L The KIDSCREEN-52 quality of life measure for children and adolescents: psychometric results from a cross-cultural survey in 13 European countries Value Health 2008 11 645 658 10.1111/j.1524-4733.2007.00291.x 18179669

[B14] Erhart M Ravens-Sieberer U Dickinson HO Colver A Rasch measurement properties of the KIDSCREEN quality of life instrument in children with cerebral palsy and differential item functioning between children with and without cerebral palsy Value Health 2009 12 782 792 10.1111/j.1524-4733.2009.00508.x 19490565

[B15] Jafari P Bagheri Z Safe M Item and response-category functioning of the Persian version of the KIDSCREEN-27: Rasch partial credit model Health Qual Life Outcomes 2012 10 127 10.1186/1477-7525-10-127 23078650 PMC3545831

[B16] Engelen V Koopman HM Detmar SB Raat H van de Wetering MD Brons P Anninga JK Abbink F Grootenhuis MA Health-related quality of life after completion of successful treatment for childhood cancer Pediatr Blood Cancer 2011 56 646 653 10.1002/pbc.22795 21298753

[B17] Bekesi A Torok S Kokonyei G Bokretas I Szentes A Telepoczki G Group TE Health-related quality of life changes of children and adolescents with chronic disease after participation in therapeutic recreation camping program Health Qual Life Outcomes 2011 9 43 10.1186/1477-7525-9-43 21672254 PMC3141371

[B18] van Dijk J Huisman J Moll AC Schouten-van Meeteren AY Bezemer PD Ringens PJ Cohen-Kettenis PT Imhof SM Health-related quality of life of child and adolescent retinoblastoma survivors in the Netherlands Health Qual Life Outcomes 2007 5 65 10.1186/1477-7525-5-65 18053178 PMC2219958

[B19] Laffond C Dellatolas G Alapetite C Puget S Grill J Habrand JL Doz F Chevignard M Quality-of-life, mood and executive functioning after childhood craniopharyngioma treated with surgery and proton beam therapy Brain Inj 2012 26 270 281 10.3109/02699052.2011.648709 22372414

[B20] Johansson E Björk O Wettergren L af Sandeberg M Health-related quality of life relates to school attendance in children on treatment for cancer J Pediatr Oncol Nurs 2008 25 265 274 10.1177/1043454208321119 18648091

[B21] Bond TG Fox CM Applying The Rasch Model Fundamental Measurement in the Human Sciences 2007 2 New York, United States of America: Routledge

[B22] Hagquist C Bruce M Gustavsson JP Using the Rasch model in nursing research: an introduction and illustrative example Int J Nurs Stud 2009 46 380 393 10.1016/j.ijnurstu.2008.10.007 19059593

[B23] Linacre JM Winsteps-Rasch Model computer program

[B24] Linacre JM Smith EV, Smith RM Otimizing Rating Scale Category Effectiveness Introduction to Rasch Measurement Theory, Models and Applications 2004 Maple Grove: JAM Press Publisher 258 278

[B25] Smith AB Rush R Fallowfield LJ Velikova G Sharpe M Rasch fit statistics and sample size considerations for polytomous data BMC Med Res Methodol 2008 8 33 10.1186/1471-2288-8-33 18510722 PMC2440760

[B26] Linacre JM A User’s Guide to Winsteps, Ministeps Rasch-Model Computer Programs. Program Manual 3.73.0 2011 http://www.winsteps.com/a/winsteps-manual.pdf23797254

[B27] Mantel N Haenszel W Statistical aspects of the analysis of data from retrospective studies of disease J Natl Cancer Inst 1959 22 719 748 13655060

[B28] Conrad KJ Smith EV Jr International conference on objective measurement: applications of Rasch analysis in health care Med Care 2004 42:I 1 6 14707750 10.1097/01.mlr.0000103527.52821.1c

[B29] Linacre JM Sample size and item calibration [or person measure] stability Rasch Measurement Transactions 1994 7:4 328 http://www.rasch.org/rmt/rmt74m.htm

[B30] Mattsson E Lindgren B Von Essen L Are there any positive consequences of childhood cancer? A review of the literature Acta Oncol 2008 47 199 206 10.1080/02841860701765667 18210296

[B31] McDougall J Tsonis M Quality of life in survivors of childhood cancer: a systematic review of the literature (2001–2008) Support Care Cancer 2009 17 1231 1246 10.1007/s00520-009-0660-0 19488790

[B32] Sam KL Wang HY Li C Lo SK Item hierarchy of the Chinese version of cerebral palsy quality of life for children Eur J Paediatr Neurol 2012 1 9 10.1016/j.ejpn.2012.06.001 22750348

[B33] Fayers PM Machin D Quality of Life: the assessment, analysis and interpretation of patient-reported outcomes 2007 Second West Sussex, England: Wiley

[B34] Berg Doukkali E Winterling J Eriksson LE Lampic C Wettergren L af Sandeberg M Adolescent and young adult views of daily life five years after a childhood cancer diagnosis. [Abstract] Pediatr Blood Cancer 2012 59 6

